# Integrating Economic Costs and Biological Traits into Global Conservation Priorities for Carnivores

**DOI:** 10.1371/journal.pone.0006807

**Published:** 2009-08-27

**Authors:** Rafael Dias Loyola, Luiz Gustavo Rodrigues Oliveira-Santos, Mário Almeida-Neto, Denise Martins Nogueira, Umberto Kubota, José Alexandre Felizola Diniz-Filho, Thomas Michael Lewinsohn

**Affiliations:** 1 Departamento de Ecologia, Instituto de Ciências Biológicas, Universidade Federal de Goiás,Goiânia, Goiás, Brazil; 2 Departamento de Ecologia, Instituto de Biologia, Universidade Federal do Rio de Janeiro, Rio de Janeiro, Rio de Janeiro, Brazil; 3 Departamento de Ecologia, Instituo de Ciências Biológicas, Universidade de Brasília, Brasília, Distrito Federal, Brazil; 4 Departamento de Zoologia, Instituto de Biologia, Universidade Estadual de Campinas, Campinas, São Paulo, Brazil; 5 Graduate Program in Ecology, Instituto de Biologia, Universidade Estadual de Campinas, Campinas, São Paulo, Brazil; University of California, Berkeley, United States of America

## Abstract

**Background:**

Prioritization schemes usually highlight species-rich areas, where many species are at imminent risk of extinction. To be ecologically relevant these schemes should also include species biological traits into area-setting methods. Furthermore, in a world of limited funds for conservation, conservation action is constrained by land acquisition costs. Hence, including economic costs into conservation priorities can substantially improve their conservation cost-effectiveness.

**Methodology/Principal Findings:**

We examined four global conservation scenarios for carnivores based on the joint mapping of economic costs and species biological traits. These scenarios identify the most cost-effective priority sets of ecoregions, indicating best investment opportunities for safeguarding every carnivore species, and also establish priority sets that can maximize species representation in areas harboring highly vulnerable species. We compared these results with a scenario that minimizes the total number of ecoregions required for conserving all species, irrespective of other factors. We found that cost-effective conservation investments should focus on 41 ecoregions highlighted in the scenario that consider simultaneously both ecoregion vulnerability and economic costs of land acquisition. Ecoregions included in priority sets under these criteria should yield best returns of investments since they harbor species with high extinction risk and have lower mean land cost.

**Conclusions/Significance:**

Our study highlights ecoregions of particular importance for the conservation of the world's carnivores defining global conservation priorities in analyses that encompass socioeconomic and life-history factors. We consider the identification of a comprehensive priority-set of areas as a first step towards an *in-situ* biodiversity maintenance strategy.

## Introduction

Conservation assessment and planning aim to optimize the allocation of scarce conservation funds by prioritizing areas for protection [1], [2]. This approach has been increasingly applied at regional [3]–[5], continental [6]–[10] and global scales [11]–[13]. Several major templates of global prioritization for biodiversity conservation were produced over the past decades [14], including the Biodiversity Hotspots and the High-Biodiversity Wilderness Areas [11], [15], the Global 200 ecoregions [12], and the Endemic Bird Areas [16]. These templates fit within the core of conservation planning theory, i.e. the conceptual framework that concerns irreplaceable and/or vulnerable areas [1]. They have, however, overlaid very distinct priorities onto this framework: some prioritize highly irreplaceable or vulnerable areas while others, conversely, favor areas with low levels of vulnerability [14].

This happens because, albeit most of these templates prioritize irreplaceable areas, some are reactive, i.e. they usually attribute high importance to areas with the highest number of threatened or endemic species or where extensive habitat loss has already taken place [11], [12], [16] (these approaches put emphasis on high vulnerability); whereas others are proactive, i.e. they put emphasis on low vulnerability aiming to protect ecosystems to avoid they become vulnerable in a foreseeable future [15]. Recent approaches have stressed the need for acting proactively as mammal species respond differently to threats [8], [17], [18] and several factors can influence such responses. They are proactive in a way that they do not prioritize species that actually happen to be threatened, but are, for distinct reasons, marching to extinction. Cardillo *et al.* [17], [19] were amongst the first to emphasize the importance of vulnerable, not yet threatened species, and proposed the use of life-history traits to infer such vulnerability. They showed that extinction risk in mammals can be driven both by environmental factors (e.g. habitat loss, climate change) and intrinsic biological traits of the species (e.g. gestation length, body size, population density). Furthermore, small and large species have different probabilities of extinction given that smaller species are primarily affected by environmental factors (including human impacts) while larger species are also constrained by their intrinsic traits.

Specifically for mammals of the order Carnivora (i.e. the carnivores), Cardillo *et al.* [20] proposed that some species are likely to move more rapidly towards extinction than others, by predicting extinction risks from their biology and combining it with projected human population density. They argued that a preventive approach to species conservation is required for protecting species that may not be threatened at present but may become so in a foreseeable future. Recently, Loyola *et al.* [9] also included species evolutionary and ecological traits in different prioritization scenarios for Neotropical mammals and were able to indicate regions that are less impacted today due to human activities while harboring most very vulnerable species.

The order Carnivora includes several major conservation icons, such as the tiger, and many other flagship, umbrella, keystone, and indicator species [21]–[23]. Their regional extinction could produce marked alterations in community composition and structure, as part of more general defaunation [24], which affects mesopredators, omnivores, and herbivores [24]. Defaunation can even modify plant population dynamics at a regional scale [25]. Some notorious carnivores, such as the jaguar in South America, also figure prominently in human-wildlife conflicts. This species may prey upon livestock, which, in turn, leads to human illegal activities (hunting, poaching, poisoning) that adversely affect their viability [26] - although not all human-carnivore conflict involves human illegal activities, as there is, for instance, legal hunting and government ‘problem animal’ control. Beyond the charismatic appeal of certain carnivores, protection for the entire group would be more effective if conservation strategies were focused on the prioritization of geographical areas or entire ecological communities, rather than addressing individual species separately [23].

In a world of limited conservation funds, prioritization of areas for conservation has often been limited by land acquisition [26], although it has been more generally limited by lack of resources, including management. Recently, Underwood *et al.* [27] argued that efficiency in prioritization would be better measured in terms of conservation returns on financial investment. There is growing that the inclusion of economic costs of conservation into prioritization analyses can lead to substantial gains in effectiveness [27], [28]. Therefore, the allocation of funds to land acquisition should be optimized in a systematic conservation planning framework, which will improve its chances of being carried out [29].

In this paper, we used broad-scale biogeographical data of carnivore species distribution - occurrence in world ecoregions [30] - to identify sets of ecoregions capable of representing all carnivore species at a global scale. To this end, we examined four conservation scenarios based on the joint mapping of economic costs and species biological traits, which (1) identify the most cost-effective sets of ecoregions, indicating best options for investments for safeguarding each carnivore species, and (2) establish sets that can maximize species representation in areas that harbor carnivores with higher extinction risks and therefore require urgent conservation action. We compared these results with a reference “null” scenario that minimizes the total number of ecoregions in the final solution, regardless of human threats and economic costs. More importantly, we also produced a combined solution in which both biological traits and economic costs were included. This scenario seeks to simultaneously maximize vulnerability across included carnivore species while minimizing land acquisition costs. Finally, we also evaluated each of these scenarios relative to their amount of area already protected, their available area for conservation and their estimated human population density in 2015. Evaluating the congruences among these conservation plans allowed us to identify where conservation is likely to yield the best return per investment at the ecoregion scale.

## Results

**Figure 1:**
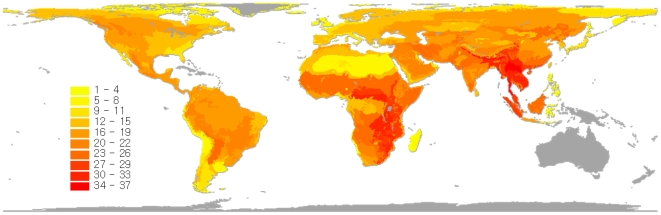
Global pattern of carnivore species richness.

Carnivore species richness is especially high in southeast Asia, the Philippines, and central and southeast Africa (Fig. 1). Other species-rich ecoregions are spread across Central America and the tropical Andes, as well as the western U.S., southern Africa, central Asia and the Middle East (Fig. 1). Ecoregions of southern South America, those in the east coast of the U.S., and those belonging to the Sahara and Arctic realms have relatively few carnivore species.

**Figure 2:**
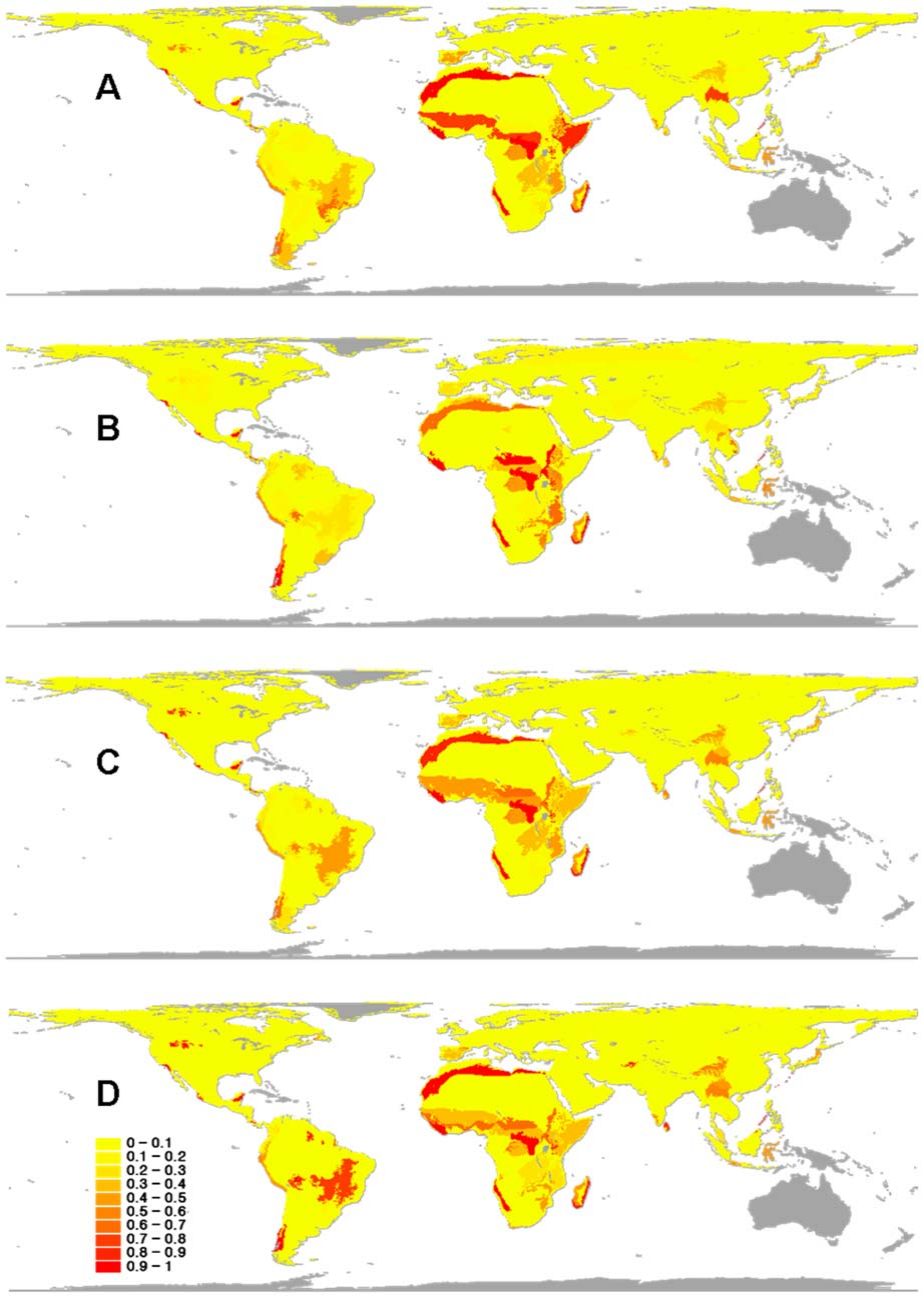
Irreplaceability patterns in the four different conservation planning scenarios. Spatial patterns of irreplaceability in the four different conservation planning scenarios: minimum ecoregion (A), cost-effective (B), highly vulnerable (C), and a combined scenario (D) that considered both species vulnerability (estimated from their biological traits) and economic costs. Irreplaceability values are the frequency of ecoregions in 100 optimal solutions for the entire 236 species of carnivores found in 661 ecoregions of the world. Values range from yellow (low) to red (high); countries in grey have no native carnivores.

Under the minimum-ecoregion scenario, only 14 ecoregions occurred in all of the 100 optimal sets that represent each species at least once and thus have maximum irreplaceability (Fig. 2A). Such areas are concentrated in a belt in central and northern Africa, but include also ecoregions in southern Africa, Madagascar and near the Himalayan Mountains (Fig. 2A). Ecoregions with irreplaceability values higher than 70% include the Yucatán moist forests in Mexico, the Valdivian temperate forests in Chile, the Argentinian Patagonian steppe, and Brazilian Cerrado, as well as ecoregions in southeast Africa.

Irreplaceability values of ecoregions selected in the cost-effective scenario were similar to those in the minimum-ecoregion set. Sixteen ecoregions occurred in all optimal solutions for this scenario: these are located in central Africa, and in certain Neotropical regions, such as the Valdivian temperate forests, the Yucatán moist forests and the Florida Everglades (Fig. 2B). Ecoregions with irreplaceability values higher than 70% are located again in Africa and southeast Asia (Fig. 2B).

Only 13 ecoregions were included in all optimal solutions for the highly vulnerable scenario for global carnivore conservation (Fig. 2C). These ecoregions occur in North America (e.g. the South Central Rockies forests, the Californian Chaparral, the Trans-Mexican Volcanic Belt pine-oak forests, and the Yucatán Moist Forests), Central America (the Talamancan Montane Forests) and Africa (e.g. the North Saharan steppe and woodlands, the East Sudanian savanna, the Northeastern Congolian forests, and the Madagascar lowland forests) (Fig. 2C).

Finally, the scenario seeking to simultaneously maximize vulnerability across included carnivore species while minimizing land acquisition costs (Fig. 2D) had 15 ecoregions included in all optimal solutions. These ecoregions are again concentrated in North America (e.g. the South Central Rockies forests, the Californian Chaparral, and the Yucatán Moist Forests), South America (the Valdivian temperate forests, in Chile), and Africa (e.g. the North Saharan steppe and woodlands, the East Sudanian savanna, and the Madagascar lowland forests).

**Figure 3:**
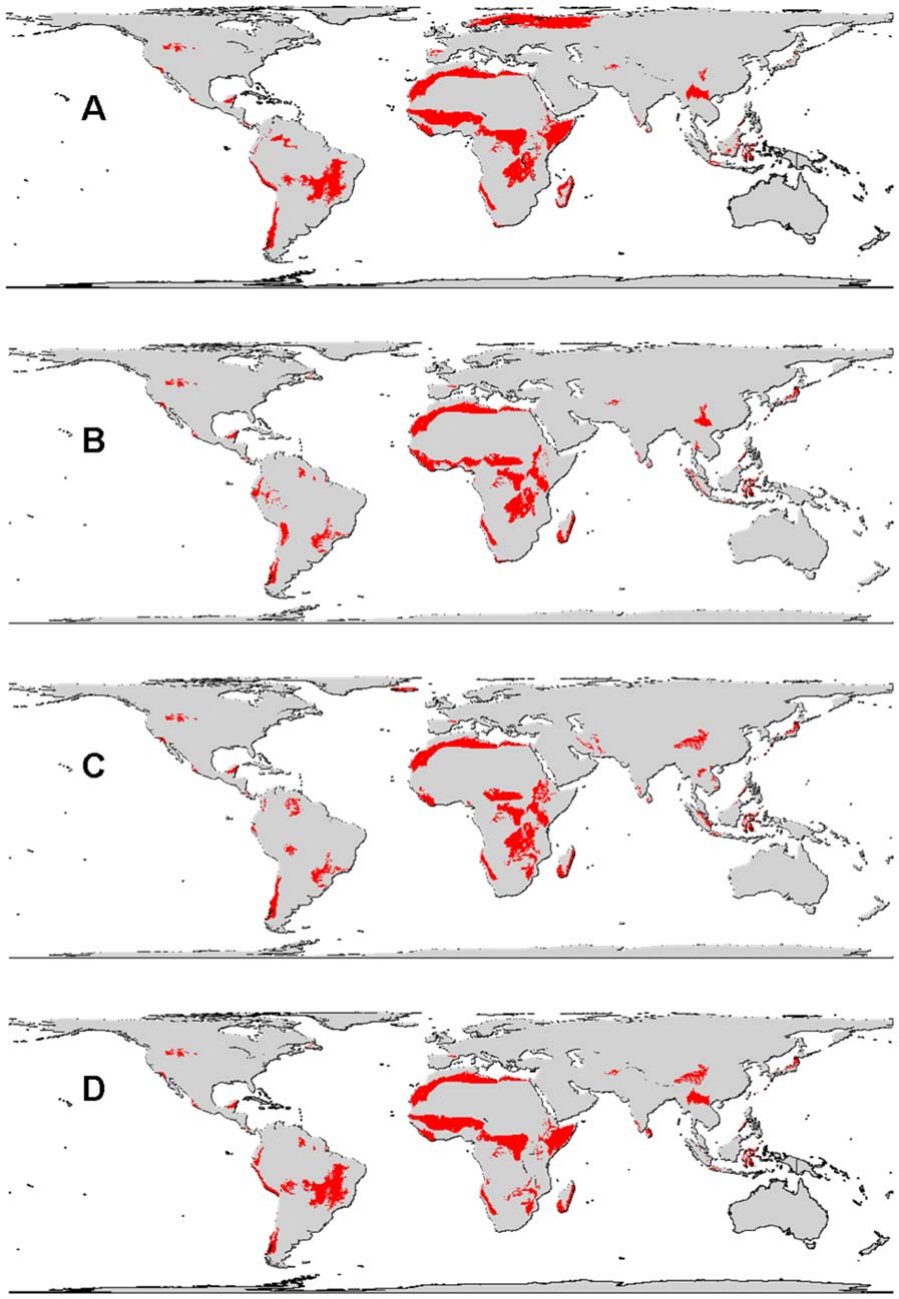
Key ecoregion sets for representing the World's carnivores in the four different conservation planning scenarios. Minimum sets of ecoregions for representation of the World's carnivores in the four different conservation planning scenarios: minimum ecoregion (A), cost-effective (B), highly vulnerable (C), and a combined scenario (D) considering both species vulnerability (estimated from their biological traits) and economic costs.

The minimum-ecoregion scenario needed 41 ecoregions to represent all carnivore species. These areas are mainly concentrated in Africa (Fig. 3A). In the cost-effective set, 44 ecoregions were able to represent all 236 species at least once (Table 1, Table S1, Fig. 3B). These ecoregions are also highly concentrated in Africa and more spread across the New World and southeast Asia, coinciding only partially with those selected under the highly vulnerable scenario and with those selected under the minimum-area scenario (Table 1, Table S1, Fig. 3B–C). The highly vulnerable scenario harbors 43 ecoregions, which are clustered primary in Africa and more widely distributed across South America and southern Asia (Fig. 3C). The combined scenario – that considered both species high vulnerability and economic costs of land acquisition simultaneously – had 41 ecoregions highly concentrated in Africa, South America and southern Asia (Fig. 3D).

**Table 1:** Summary results for the four systematic planning scenarios for conservation of the world's carnivores.

Conservation goal	Conservation scenario							
	Minimum ecoregion		Cost effective		Highly vulnerable		Combined (vulnerability+costs)	
**N° Ecoregions**	41		44		43		41	
**N° highly irreplaceable ecoregions**	14		16		13		15	
**Total area** (×10,000 km^−2^)	126,75		903,09		867,10		1134,85	
**Mean land cost** (×1000 US$ km^−2^)	980,12	(±2039.69)	782,28	(±2039.69)	962,41	(±2033.11)	932,33	(±2087.97)
**Mean proportion of protected area**	0,17	(±0.21)	0,15	(±0.21)	0,16	(±0.21)	0,17	(±0.22)
**Mean proportion of land-use area**	0,31	(±0.26)	0,36	(±0.27)	0,31	(±0.27)	0,30	(±0.27)
**Mean proportion of available area**	0,53	(±0.28)	0,50	(±0.28)	0,55	(±0.28)	0,56	(±0.29)
**Mean human population density in 2015** (people km^−2^)	6,28	(±17.61)	5,72	(±16.94)	6,54	(±17.36)	4,56	(±10.35)

Numbers in parenthesis indicate SD. Highly irreplaceable ecoregions mean those with 100% inclusion across runs.

The mean predicted population density in 2015 was higher in the highly vulnerable conservation scenario (Table 1). The minimum-ecoregion set spanned a much larger total area than other scenarios. Relative to the mean proportion of area under protection or available for conservation, the three scenarios were very similar (Table 1). The cost-effective scenario presented a higher mean value of land use than the others, albeit the difference was fairly small. Finally, the combined scenario revealed a key set of 41 ecoregions, of which 15 have high irreplaceability values (Table 1). This last scenario should be considered the most important one as it considered at the same time those ecoregions needing urgent intervention for carnivore conservation while forcing the inclusion ecoregions that fit into the most cost-effective planning scenario (Fig. 3D). It has also the lowest estimate of population density in 2015.

## Discussion

Recently, several studies have portrayed priority areas for conservation of various taxonomic groups at different spatial scales [8]–[10], [13], [26], [31], [32]. However, very few focused on carnivores [9], [21], [22]. Our study highlights ecoregions of particular importance for the conservation of the world's carnivores, and indicates global conservation priorities for these vertebrates explicitly incorporating land acquisition costs as a key socioeconomic factor, as well as variation in extinction risk based on relevant biological traits. Our selection procedure produces several options for areas where conservation of carnivores should be focused. Choice of particular high-priority ecoregion set should then depend on the priorities adopted in a general conservation policy.

A growing body of evidence indicates that large-bodied species, with sizeable home ranges that occur at low densities and feed at higher trophic levels, are more prone to local extinction in habitat fragments [17], [19], [33], [34]. This seems to be the case for many if not most carnivores. As pointed out by Cardillo *et al.* [20], small geographic ranges, low population densities, and low litter sizes are traits that limit the maximum population size a species can attain. Gestation length and interbirth interval determine population resilience, that is, how quickly populations can recover from low levels [35]. Moreover, their need for large foraging areas, and dependence on prey species that may themselves be in jeopardy [36] put carnivores in danger around the globe, particularly in regions where human population density is high [20]. This reinforces the importance of including species biological traits into conservation planning analyses [8], [9].

The disparity in economic cost among ecoregions means that there is potential for great benefit in seeking efficient financial investments [27]. Area-setting analyses that neglect cost implicitly assume that this factor is homogeneously distributed across the geographic space, which may reduce priority-set efficiency. Note that our results clearly indicate that an optimal set under minimum-ecoregion criterion was less efficient, in terms of total area and economic costs, than the other two scenarios (see Table 1). That means it is possible to cover more ecoregions with less area when considering economic costs, as several “cheap” ecoregions are small and concentrated in Africa. In a recent essay, Bode *et al.* [13] concluded that the inclusion of socioeconomic factors (threat and cost) is crucial for determining priorities for biodiversity conservation. They created efficient global funding schedules using information about costs, species-endemism level, and predicted habitat loss rates in the biodiversity hotspots proposed by Mittermeier *et al.* [11]. More important, they found that funding allocations were less sensitive to choice of taxon assessed, than to variation in cost and threat. These results strengthen confidence in global-scale decisions guided by single taxonomic groups [13]. Consequently, our combined scenario (Fig. 2D and 3D) is of high potential relevance for effective conservation strategies of the world's carnivores. Actually, we suggest that our combined scenario should have prominence in this study as it conveyed a low total number of ecoregions, high proportion of irreplaceable ecoregions, relatively low land acquisition costs, high proportion existing protected area, high available area, and low HPD (Table 1).

The priority sets identified in this study complement and lend support to other priority-setting frameworks [14]. Important areas consensually indicated as priority for carnivores are mainly in the U.S. [22], Mexico [9], [21], Tropical Andes, Brazilian Atlantic forest, and southern South America [9]. Other congruences were also observed with priority areas proposed for wider taxonomic groups such as mammals and amphibians in Africa [31], threatened anurans in the Neotropics [8], [37], terrestrial vertebrates both in the Neotropics [10] and worldwide [12], as well as endemic plants at global scale [11], [12].

The necessity of developing conservation action at the landscape level – by itself or combined with broad-scale actions [34] – supports the use of ecoregions as fundamental geographic units. We chose to use ecoregions because these broad areas are defined according to physiographic and biotic features and, therefore, should reflect biogeographic boundaries more closely than political topographical entities. They are also less sensitive to heterogeneity in distribution data than grid-based analyses [38] and are being employed by major conservation organizations as well as many government agencies [5], [9], [12] – although an ecoregion approach entails its own caveats [5], [8], [10]. Hence, broad-scale area assessments provide frameworks within which finer-scaled options for conservation setting and resource allocation have to be established and analyzed [14].

Protected areas are the keystone of current conservation strategies. Our results showed that mean percentage of protected area in ecoregions sets selected by different conservation scenarios vary between 14 to 17%. However, there is also great variation among individual ecoregions attaining 38% of protection whereas others have none. We should highlight the relative high proportion (>0.56) of area still available for conservation in the our combined set – which, coupled with the lowest estimate of population density in 2015, may offer concrete opportunities for designing and establishing protected areas in several key regions. Note that our analyses include average estimated land costs and human population density as socioeconomic factors, but there are clearly other cultural, economic, and political concerns which affect such policies. Furthermore, conservation implementation and outcomes are also affected by many complex interacting socioeconomic forces like those related to governance, institutional capacity, and dynamic markets.

To conclude, we must acknowledge that prioritization analyses such as these ones should be considered more indicative than prescriptive. It should be considered by conservation planners as a rapid and coarse assessment of potential costs in achieving a particular conservation goal [21]. The identification of a comprehensive set of natural areas is only a first step towards an *in-situ* biodiversity maintenance strategy, which is part of a much more complex process of policy negotiation and implementation [8]. Final decisions should be based on comparing alternatives and involving different institutions [39]. Our scenarios are no substitute for this negotiation process, but they are part of a wide-ranging effort [11], [40] to strengthen the scientific basis for conservation decisions.

## Materials and Methods

### Data

We used the WWF hierarchical classification of ecoregions [30], [41]. The database used for the analyses contains the current species list of mammals in all terrestrial ecoregions. We focused our analyses on the entire worldwide set of 236 carnivore species (occurring in 661 ecoregions), whose occurrence ranges were obtained from Wilson & Reeder [42], which we also followed carnivore taxonomy. Information on updates, detailed descriptions of the database, and complete lists of sources can be obtained from the WWF [30].

For each species, we obtained five biological variables used by Purvis *et al.* [43] and updated from Cardillo *et al.* [20], to include more recently published information. These variables were species body size, interbirth interval, litter size, gestation length, and population density. We selected these variables because they were immediately available from literature and have been used as indicators of extinction risk for mammals and carnivores, in particular [17], [19], [20], [43]. We also excluded those that convey the same information as they were intercorrelated (e.g. body mass and body length).

Balmford *et al.* [44] found that land acquisition costs are closely related to annual recurrent management costs [national mean land acquisition costs km^−2^ were 50.6±13.5 (mean±SE) times national mean recurrent costs km^−2^ y^−1^]. Following Underwood *et al.* [27], we calculated the cost of acquiring land for protection by first applying an equation for the regular cost of annual management – originally proposed by Balmford *et al.* [44] – and then multiplying the values found by the correction factor of 50.6 [27], [44] to estimate the cost of land acquisition in each ecoregion. Underwood *et al.*'s [27] formula for land acquisition for conservation was modified to:
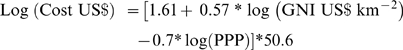



Where GNI is Gross National Income, and PPP is Purchasing Power Parity. We excluded an additional term for the influence of reserve size on annual management cost [27], because ecoregions cannot be conserved in their entirety [5], [8]. Since our objective was to identify priority sets among all possible sets of ecoregions, a relative monetary value per unit area per ecoregion was used for comparison. We obtained GNI from the International Monetary Fund's International Financial Statistics [45] and compiled PPP and GDP deflators from the World Bank (http://devdata.worldbank.org/wdi2006/contents/Section4.htm). As the PPP term is the PPP conversion factor divided by the exchange rate, we calculated the area-weighted average after determining the costs for each country to allow the inclusion of ecoregions that span multiple countries.

Finally, we obtained the following data for each ecoregion from WWF [30]: total area (in km^2^), proportion of area protected (area under IUCN category I-VI), proportion of land-use area (area under agricultural lands and urbanization) and proportion of land available for conservation [calculated as the total area – (land-use area+protected area)]. For our measures of Human Population Density (HPD), we used the Gridded Population of the World [46], a spatially explicit global database of predicted HPD for 2015, coarsened to a resolution of 0.5×0.5°. HPD was calculated from GPW specifically for each ecoregion (i.e. based on mean grid cells values falling within ecoregion).

### Analyses

We set up four different conservation-planning scenarios all of them trying to resolve the optimization problem know as “the set-covering problem”. This mathematical selection method aims to represent all natural features (e.g. species or habitats) a given number of times in the smallest possible area, fewest numbers of sites, or with the lowest overall cost. Usually, analyses of this type have concentrated on the identification of the minimum set of sites required to represent all species at least once [2]. This was done here, i.e. our conservation goal was to represent every carnivore species in at least one ecoregion of the world, for each of the following conservation scenario. A simulated annealing algorithm was used to achieve this. It begins with a random set of ecoregions and, for each iteration, swaps ecoregions in and out of that set, measuring the change in cost according to a cost function. The optimization procedure was repeated 100 times, and final conservation scenarios were obtained after 20 million iterations, implemented in the Site Selection Mode routine of the software SITES [47], [48]. We also set a high penalty value for losing a species (value 3, in SITES *species.dat* file), so that the final solution for each conservation scenario included all species.

The site-selection procedure was limited by different constraints operating at each conservation scenario. The minimum-ecoregion scenario (A) was a reference “null” scenario aimed at the representation of all species in the minimum number of ecoregions in the world; threats to species and economic cost of each ecoregion were not considered. This means that the site-selection algorithm had to find solutions that represent all species minimizing the number of ecoregions. As several solutions were tied for number of ecoregions, we chose the one with the smallest total area. Thus, this scenario minimizes the number of ecoregions and the area targeted for high-priority conservation action.

In the cost-effective scenario (B), all species were represented whereas the economic cost of each ecoregion was equaled to the calculated cost (US$ km^−2^) of land acquisition. This means that the site-selection algorithm had to find solutions that represent all species minimizing the mean acquisition cost per ecoregion in the set of high priority for conservation. Therefore, we could find the “cheapest” scenario among several options for global carnivore conservation within a macroecological framework.

**Figure 4:**
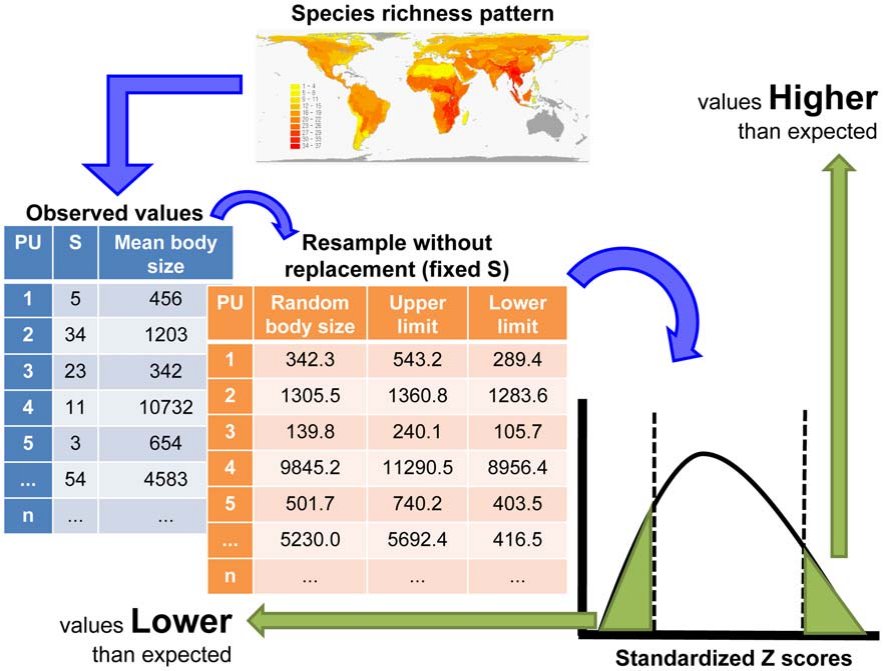
General randomization procedure used to generate vulnerability costs based on species biological traits. This was done this way: for each ecoregion we calculated the mean value of a particular biological trait (e.g. body size) based on the species occurring there – see the pattern of species richness on the upper part of the figure. Then, we resampled without replacement the same number of species found in the ecoregion from the species pool in order to calculate an expected mean value for each biological trait included in this study. This was done 1000 times, and the expected distributions of trait values were compared with those actually observed within each ecoregion. We were then able to evaluate if a given ecoregion had trait values higher or lower then we would expect if species were able to occupy all the geographical space. These values were submitted to a statistical z transformation in order to be compared and analyzed together.

In highly vulnerable scenario (C), we aimed to find a minimum set of ecoregions containing a greater proportion of species whose biological traits predispose them to greater extinction risk. To produce this set, we attributed a vulnerability cost for each ecoregion based on the biological variables mentioned above. We calculated mean values for these species' traits within each ecoregion and identified ecoregions in which trait values were higher or lower than expected from a null-model of equiprobable species occurrence in all ecoregions, given the observed richness found in an ecoregion (see Fig. 4). This was done this way: for each ecoregion we calculated the mean value of a particular biological trait (e.g. body size) based on the species occurring there. Then, we resampled without replacement the same number of species found in the ecoregion from the species pool in order to calculate an expected mean value for each biological trait included in this study. This was done 1000 times, and the expected distributions of trait values were compared with those actually observed within each ecoregion. We were then able to evaluate if a given ecoregion had trait values higher or lower then we would expect if species were able to occupy all the geographical space (Fig. 4). Randomizations were performed in BootRMD software written by one of us (JAFDF) in Visual Basic® and available from the authors upon request. Trait values were standardized (submitted to a *z* statistical transformation generating *z*-scores) to allow comparison and calculations among ecoregions. The *z*-scores representing each variable within ecoregions were summed, so that “very vulnerable” ecoregions for conservation are those that tend to aggregate carnivore species with larger bodies, higher interbirth intervals, longer gestation periods, lower litter sizes, and smaller local populations [9]. In this scenario the site-selection algorithm had to find solutions that represent all species favoring ecoregions in which these biological traits have values higher than expected, as explained above. These ecoregions are highly vulnerable because they capture species whose biological traits predispose them to greater extinction risk under the ensuing conservation plan. We did not use other risk indices, such as the IUCN categories, because we sought for species that could not be actually threatened at this time, but to which attention should be paid as their biological traits intrinsically predispose them to extinction.

Finally, we combined in the last scenario all variables related to species biological traits as well as economic costs associated with land acquisition within ecoregions to produce an optimal combined scenario (D) capable of representing all carnivore species while favoring the inclusion of ecoregions with maximum vulnerability and lower mean economic costs, whenever possible. To use both biological traits and economic costs as constraints in such prioritization analysis, we performed the same calculation of *z*-scores described above, including *z*-scores for land acquisition costs. This means that mean economic costs were calculated for each ecoregion and then were shuffled assuming that costs are not geographically structured, i.e. ecoregion costs could vary randomly. We calculated *z*-scores for land acquisition costs indicating if an ecoregion has costs that are higher or lower than expected by chance. In the combined scenario, ecoregion vulnerability and cost had the same weight; otherwise they could not be compared nor summed. These *z*-scores were summed and used with those indicating species extinction risk as used as constraints to produce this combined set. This approach has been called an “iterative-stage protocol” in multi-criteria conservation planning analyses [2].

Because often there are multiple combinations of ecoregions that satisfy the representation goal in each conservation scenario, we also integrated such alternative solutions into a map in which the relative importance of each ecoregion is indicated by its rate of recurrence in optimal subsets (see Fig. 2B–D). This is also an estimate of the irreplaceability of ecoregions, ranging from 0 to 1. Although there are multiple combinations for satisfying each representation goal, we were able to identify a consensus solution for each scenario in which total ‘costs’ were the smallest. These optimal solutions were used to indicate priority sets of ecoregions for carnivore conservation at a global scale (see also Loyola *et al.* [8]–[10]).

The summary results of each systematic planning scenario were evaluated according to their total amount of area (in km^2^), total number of ecoregions, mean land acquisition costs, proportion of protected area, proportion of land-use area, and proportion of available area for conservation, as well as their predicted HDP in 2015 – a measure of indirect conservation conflict *sensu* Cardillo *et al.* [20]. This *a posteriori* comparison of conservation planning scenario has been called a “terminal-stage protocol” in multi-criteria conservation planning analyses [2].

Finally, the spatial pattern in carnivore species richness, as well as the priority sets of ecoregions obtained from our analyses, were overlaid in a map of World ecoregions [41] using ArcView GIS 3.2 (ESRI, Redmond, California). Shapefiles and associated attribute tables were obtained from WWF [30]. We employed an equal-area cylindrical projection in all maps.

## Supporting Information

Table S1 Priority ecoregions for conserving the World's carnivores included (indicated by “1”) in optimal sets under a minimum-ecoregion scenario, a cost-effective scenario, a highly vulnerable scenario, and a combined scenario - along whit their irrepleceability values. Ecoregion area obtained from [29].
(1.33 MB XLS)

## References

[1] Margules CR, Pressey RL (2000). Systematic conservation planning.. Nature.

[2] Margules CR, Sarkar S (2007). Systematic conservation planning..

[3] Cowling RM, Pressey RL, Rouget M, Lombard AT (2003). A conservation plan for a global biodiversity hotspot - the Cape Floristic Region, South Africa.. Biol Conserv.

[4] Smith RJ, Goodman PS, Matthews WS (2006). Systematic conservation planning: a review of perceived limitations and an illustration of the benefits, using a case study from Maputaland, South Africa.. Oryx.

[5] Loyola RD, Kubota U, Lewinsohn TM (2007). Endemic vertebrates are the most effective surrogates for identifying conservation priorities among Brazilian ecoregions.. Divers Distrib.

[6] Dinerstein E (1995). A conservation assessment of the terrestrial ecoregions of Latin America and the Caribbean..

[7] Burgess DN (2004). Terrestrial ecoregions of Africa and Madagascar: a conservation assessment..

[8] Loyola RD, Becker CG, Kubota U, Haddad CFB, Fonseca CR (2008a). Hung out to dry: choice of priority ecoregions for conserving threatened Neotropical anurans depends on life-history traits.. PLoS ONE.

[9] Loyola RD, Oliveira G, Diniz-Filho JAF, Lewinsohn TM (2008b). Conservation of Neotropical carnivores under different prioritization scenarios: mapping species traits to minimize conservation conflicts.. Divers Distrib.

[10] Loyola RD, Kubota U, Fonseca GAB, Lewinsohn TM (2009). Key Neotropical ecoregions for conservation of terrestrial vertebrates.. Biodivers Conserv.

[11] Mittermeier RA, Robles-Gil P, Hoffman M, Pilgrim J, Brooks T (2004). Hotspots revisited: Earth's biologically richest and most endangered terrestrial ecoregions..

[12] Olson DM, Dinerstein E (2002). The Global 200: Priority ecoregions for global conservation.. Ann Miss Bot Gard.

[13] Bode M, Wilson KA, Brooks TM, Turner WR, Mittermeier RA (2008). Cost-effective global conservation spending is robust to taxonomic group.. Proc Natl Acad Sci USA.

[14] Brooks TM, Mittermeier RA, da Fonseca GAB, Gerlach J, Hoffmann M (2006). Global biodiversity conservation priorities.. Science.

[15] Mittermeier RA, Mittermeier CG, Brooks TM, Pilgrim JD, Konstant WR (2003). Wilderness and biodiversity conservation.. Proc Natl Acad Sci USA.

[16] Stattersfield AJ, Crosby MJ, Long AJ, Wege DC (1998). Endemic bird areas of the world: priorities for biodiversity conservation..

[17] Cardillo M, Mace GM, Gittleman JL, Purvis A (2006). Latent extinction risk and the future battlegrounds of mammal conservation.. Proc Natl Acad Sci USA.

[18] Becker CG, Loyola RD (2008). Extinction risk assessments at the population and species level: implications for amphibian conservation.. Biodiv Conserv.

[19] Cardillo M, Mace GM, Jones KE, Bielby J, Bininda-Emonds ORP (2005). Multiple causes of high extinction risk in large mammal species.. Science.

[20] Cardillo M, Purvis A, Sechrest W, Gittleman JL, Bielby J (2004). Human population density and extinction risk in the world's carnivores.. PLoS Biology.

[21] Valenzuela-Galván D, Vázquez LB (2008). Prioritizing areas for conservation of Mexican carnivores considering natural protected areas and human population density.. Anim Conserv.

[22] Valenzuela-Galván D, Arita HT, Macdonald DW (2008). Conservation priorities for carnivores considering protected natural areas and human population density.. Biodivers Conserv.

[23] Gittleman JL, Funk SM, MacDonald DW, Wayne RK (2001). Carnivore conservation..

[24] Dirzo R, Miranda A, Price P, Lewinsohn TM, Fernandes GW, Benson WW (1991). Altered patterns of herbivory anddiversity in the forest understory: a case study of the possible consequences of contemporary defaunation.. Plant-animal interactions: Evolutionary ecology in tropical and temperate regions.

[25] Laurance WF, Peres CA (2006). Emerging threats to tropical forests..

[26] Rondinini C, Boitani L (2007). Systematic Conservation Planning and the Cost of Tackling Conservation Conflicts with Large Carnivores in Italy.. Conserv Biol.

[27] Underwood EC, Shaw MR, Wilson KA, Kareiva P, Klausmeyer KR (2008). Protecting Biodiversity when Money Matters: Maximizing Return on Investment.. PLoS ONE.

[28] Naidoo R, Balmford A, Ferraro PJ, Polasky S, Ricketts TH (2006). Integrating economic costs into conservation planning.. Trends Ecol Evol.

[29] Davis FW, Costello C, Stoms D (2006). Efficient conservation in a utility-maximization framework.. Ecol Soc 11.

[30] [WWF] World Wildlife Fund (2006). WildFinder: online database of species distributions, version Jan-06.. http://www.worldwildlife.org/WildFinder.

[31] Rondinini C, Stuart S, Boitani L (2005). Habitat suitability models and the shortfall in conservation planning for African vertebrates.. Conserv Biol.

[32] Das A, Krishnaswamya J, Bawaa KS, Kirana MC, Srinivasc V (2006). Prioritisation of conservation areas in the Western Ghats, India.. Biol Conserv.

[33] Laurance WF, Lovejoy TE, Vasconcelos HL, Bruna EM, Didham RH (2002). Ecosystem decay of Amazonian forest fragments: a 22-year investigation.. Conserv Biol.

[34] Boyd C, Brooks TM, Butchart SHM, Edgar GJ, da Fonseca GAB (2008). Spatial scale and the conservation of threatened species.. Conserv Lett.

[35] Gittleman JL, Dunstone N, Gorman ML (1993). Carnivore life histories: A reanalysis in the light of new models.. Mammals as predators.

[36] Carbone C, Gittleman JL (2002). A common rule for the scaling of carnivore density.. Science.

[37] Urbina-Cardona JN, Loyola RD (2008). Applying niche-based models to predict endangered-hylid potential distributions: are Neotropical protected areas effective enough?. Tropical Conservation Science.

[38] Lamoreux JF, Morrison JC, Ricketts TH, Olson DM, Dinerstein E (2006). Global tests of biodiversity concordance and the importance of endemism.. Nature.

[39] Pressey RL, Possingham HP, Day JR (1997). Effectiveness of alternative heuristic algorithms for identifying indicative minimum requirements for conservation reserves.. Biol Conserv.

[40] Soutullo A, De Castro M, Urios V (2008). Linking political and scientifically derived targets for global biodiversity conservation: implications for the expansion of the global network of protected areas.. Divers Distrib.

[41] Olson DM, Dinerstein E, Wikramanayake ED, Burgess ND, Powell GVN (2001). Terrestrial ecoregions of the worlds: A new map of life on Earth.. Bioscience.

[42] Wilson DE, Reeder DM (2005). Mammal species of the World: A taxonomic and geographic reference..

[43] Purvis A, Gittleman JL, Cowlishaw G, Mace GM (2000). Predicting extinction risk in declining species.. Proc Royal Soc London B.

[44] Balmford A, Gaston KJ, Blyth S, James A, Kapos V (2003). Global variation in terrestrial conservation costs, conservation benefits, and unmet conservation needs.. Proc Natl Acad Sci USA.

[45] International Monetary Fund (2004). International Financial Statistics. Washington DC, International Monetary Fund..

[46] CIESINIFPRIWRI (2005). Gridded Population of the World (GPW).. http://sedac.ciesin.columbia.edu/plue/gpw.

[47] Andelman S, Ball I, Davis F, Stoms D (1999). SITES v. 1.0: An analytical toolbox for designing ecoregional conservation portfolios..

[48] Possingham H, Ball I, Andelman S, Ferson S, Burgman M (2000). Mathematical methods for identifying representative reserve networks.. Quantitative Methods for Conservation Biology.

